# Evaluation of ethylene oxide, gamma radiation, dry heat and autoclave sterilization processes on extracellular matrix of biomaterial dental scaffolds

**DOI:** 10.1038/s41598-022-08258-1

**Published:** 2022-03-11

**Authors:** Luciana Aparecida de Sousa Iwamoto, Mônica Talarico Duailibi, Gerson Yoshinobu Iwamoto, Débora Cristina de Oliveira, Silvio Eduardo Duailibi

**Affiliations:** 1 CTCMOL, UNIFESP-São Paulo, São Paulo, SP Brazil; 2 LMMM, UNIFESP-Diadema, São Paulo, SP Brazil

**Keywords:** Translational research, Materials science

## Abstract

Scaffolds used to receive stem cells are a promising perspective of tissue regeneration research, and one of the most effective solutions to rebuild organs. In the near future will be possible to reconstruct a natural tooth using stems cells, but to avoid an immune-defensive response, sterilize the scaffold is not only desired, but also essential to be successful. A study confirmed stem cells extracted from rat’s natural teeth, and implanted into the alveolar bone, could differentiate themselves in dental cells, but the scaffold’s chemistry, geometry, density, morphology, adherence, biocompatibility and mechanical properties remained an issue. This study intended to produce a completely sterilized dental scaffold with preserved extracellular matrix. Fifty-one samples were collected, kept in formaldehyde, submitted to partial demineralization and decellularization processes and sterilized using four different methods: dry heating; autoclave; ethylene-oxide and gamma-radiation. They were characterized through optical images, micro-hardness, XRD, EDS, XRF, SEM, histology and sterility test. The results evidenced the four sterilization methods were fully effective with preservation of ECM molecular arrangements, variation on chemical composition (proportion of Ca/P) was compatible with Ca/P proportional variation between enamel and dentine regions. Gamma irradiation and ethylene oxide presents excellent results, but their viability are compromised by the costs and technology’s accessibility (requires very expensive equipment and/or consumables). Excepted gamma irradiation, all the sterilization methods more than sterilizing also reduced the remaining pulp. Autoclave presents easy equipment accessibility, lower cost consumables, higher reduction of remaining pulp and complete sterilization, reason why was considered the most promising technique.

## Introduction

The tooth’s design is a marvel of engineering, as it is capable to absorb static and dynamic energies^1^, the dental tissues are subject to large abrasive efforts compressive and shearing forces near 700 N^2,3^. Considered the most mineralized tissue of the human body, enamel is composed basically of crystalline PAH (Hydroxyapatite—near 90%) in structure Ca_10_(PO4)_6_(OH)_2,_ water and organic matter (protein and lipids). The hardness of the enamel is between 3.2 and 4.4 GPa or 270 to 360 VHN and in relation to the fatigue strength it is considered friable, usually presenting micro fractures by stresses beyond its elastic limit. Tensile strength is close to 10 MPa and compressive strength is approximately 262 MPa. Its modulus of elasticity in compression tests reaches 33.6 GPa^2,4^. Dentin is a biological composite of a collagen matrix filled with nanometric apatite crystallites deficient in Ca (calcium), rich in carbonate, F (iron) and Mg (magnesium), and scattered on parallel hollow cylinders called canaliculated or dentinal tubules^3^. The dentin has tensile strength is 50 MPa and hardness from 50 to 60 VHN ^4^.

Tissue engineering is one of the most challenging areas in regenerative medicine, more than replacing damaged tissues, it is expected the restoration, maintenance or improvement of the regenerated tissue^5^. To determine the suitability of scaffolds, it is important to consider: biocompatibility, biodegradability, mechanical properties, scaffold architecture, and manufacturing technology. The recovery of organic functions, through the use of stem cells, constitute the most challenging task, when viable, the ideal substitute should be from autologous origin^6^.

Scaffolds can be produced from biological, synthetic or composite materials, the natural materials are more biologically active, usually promote excellent cell adhesion, growth, and their biodegradability allows to host cells, consequently, stimulate the production of its’ own ECM (Extracellular Matrix). The use of synthetic materials as polymers and ceramics are growing significantly, the polymers can be tailored built and biodegradation can be controlled through the composition, although still exist the risk of infection, due to reduced bioactivity. It is growing the use of polymer or ceramic based prosthesis, for example knees or shoulders, metal-ceramic is also present on teeth’s restoration or dental crowns^7–9^. Although ceramic scaffolds exhibit excellent biocompatibility, due to similarity with hydroxyapatite (HA), the applications are limited by the brittleness and difficulty to sharp, in addition new bone formed in a porous HA cannot sustain the mechanical loading needed for remodeling or the compressive forces on chewing (near 700 N)^10^. These difficulties in terms of structure, functionality and mechanical properties, when producing scaffolds from single phase biomaterial, encouraged research with composite materials, as ceramic-polymer or metal-ceramic based scaffolds^11–13^.

The scaffolds, being tridimensional structures similar to original tissue in texture, macro and micro-geometry, make easier the cellular adhesion and consequent expression, the ECM can influence the cell’s behavior, and the mechanical properties are critical to mechanic transduction, development, differentiation and regeneration^10,11,14,15,16^. The bioscaffolds can be used in tissue regeneration with ability to carry out cellular activities, drugs and genes, produced with biocompatible material, absorbable or not. As the structure is very complex, being composed by solid, fiber and gel, the best scaffold would be the natural one, because it was already able to keep living cells in activity^17^. Gupte and Ma^18^ defend the materials should bio-mimic scaffolds’ features, developing characteristics for cell-bearing and create an environment to improve cell adhesion, proliferation, differentiation and also to promote the tissue regeneration. More than design, the mechanical integrity and functionality, morphology and surface compatibility are also essential for cell adhesion, differentiation, and integration with surrounding natural tissues. Additional care need to be taken with sterilization, to avoid infection and rejection^19–21^.

To produce a complete tooth from stem cell remains a challenging task, however, the production of collagen sponges and gels, used for tooth regeneration, has shown great compatibility to retain cells and support proliferation, differentiation, and also stimulate the formation of calcified tissues^22^; the drivability of human dental stem cell to immature osseous tissues, including tooth beak of chick embryo^23^ can be a promising technique. Another well succeeded experiment, using dental pulp stem cells seeded on collagen scaffolds (in vitro), were able to produce a fresh stemmed pulp tissue^24,25^.

The idea of producing a natural tooth from stem cell has been tried seeding stem cells on a rat^16^, being partially successful on the production of a teeth. Although there was a visible separation of each layer (enamel, dentin, cement and pulp) the shape remained an issue, incentivizing the study of sterilized teeth as a bioscaffold, what supposedly would improve the results. The total cells removal from a tissue or an organ (decellularization) is a complex process, as there are structural and functional proteins constituting the ECM, if it is damaged, can compromise the adequate expression of stem cells. If used in excess of time or concentration, the demineralization and decellularization solutions may interfere on ECM and alter its ultra-structure, on the other hand, if biological contents are not completely removed, can cause an inflammatory or immune response. The pulp removal could be done through an endodontic access (perforation), but this method was not used in order to keep the ECM preserved.

Although good results are reached developing mineralized dentin, enamel and crown through the use of tissue engineering, the complex structure and interactions between the epithelium and mesenchyme tissues justify the difficulty to develop a viable organ from stem cells^5^. Lack of vascularization in scaffolds and tissue engineered constructs is a major problem^26,27^, an alternative to vascularization is engineering microvasculature by stem cells previously to implantation^28,29^.

Decellularized tissues are extensively used to obtain biomaterial scaffolds, where the efficiency of cells removal depends of the origin and the methods used to sterilize it. The ideal conditions to use these tissues include the minimum interference on biochemical composition, ultra-structure and the mechanical behavior of ECM^30,31^. The host's reaction to a foreign body can be inflammatory response or an immediate and immune rejection, being one of the reasons the use a biomaterial scaffolds, obtained through decellularization of tissues and organs, should provide better results. The conservation of ECM are well tolerated by xenogeneic receptors, to use biomaterial scaffolds, but it is recommended an adequate sterilization process to be implanted^32^.

The organ to be sterilized drives the sterilization method choice. The sterilization is a key step on biomaterials procedures, infections could cause rejection of scaffold or eventually the death of patient*.* The elimination of microorganisms as bacteria, spores, protozoa, fungi, and virus are essential to reach an acceptable level of safety on sterilization^17^. Although exist several different methods, were chosen 4 methods well known^5,10,33–36^ to be compared in this study. The autoclave sterilization (AS) is the most used in dental clinics, although the thermo-sensitivity of several hospital materials do not allow the generic use of this method^33^. In the past it was common to use the dry heat (DH) method, which uses high temperatures placing samples inside an oven, although it could promote the hydrolysis and/or the matrix fusion, compromising the biocompatibility of some organs, similarly to autoclave, this sterilization method do not affect dental samples. An alternative method could be the chemical gas sterilization through ethylene oxide (EO), this method is very effective at low temperatures, however, the gas residues are alkylation agents, what can modify and/or compromise the properties of the biomaterial^37^, reason why the mechanical properties, appearance (color and shape) were checked before and after the procedures. Finally, the ionizing gamma irradiation (GAMMA), is an effective sterilization method, with perspective of also eliminate Sars-Cov2 with the highest potential for guaranteeing the safety of patients receiving skin, amniotic membranes and bone transplants^34^.

The restoration of tooth loss is a great challenge in dentistry, and the search for a biological substitute envisages new therapeutic horizons. There are several mechanisms for demineralization, decellularization and sterilization of the organs, but was not found a definitive process for the dental scaffold. Dentistry aims not only on restoration but also on its regeneration, justifying the need of advancing on studies to mimic the teeth enabling it as biomaterial scaffold, what also will demand an efficient sterilization process.

In the first phase of this study^31^, after partial demineralization-decellularization, presented remaining pulp on almost 30% of the samples. Would be possible to reach 100% of pulp removal, using higher concentration or time extension of exposition to demineralization solution, an alternative could be an endodontic intervention to access the pulp chamber, both avoided because they could interfere on the samples’ structural integrity. failure rates because of bacterial colonization are still high The existence of remaining pulp could provide conditions to cell proliferation^38^ and represent a considerable risk of rejection or inflammatory response^32,39^, reason why the sterilization methods were introduced in this phase of the study.

The aim of this study was to obtain a viable dental scaffold, with preservation of structural properties, were compared commonly used sterilization methods (AS and DH) and high efficiency techniques (Gamma and EO), Mechanical resistance (micro-hardness), preservation of layers (enamel, dentine, cement and pulp chamber were analyzed and also the sterilization efficacy of the four methods proposed and analyze their efficacy to sterilize extracted natural teeth, enabling them as biomaterial scaffolds to posterior reception of stem cells.

## Materials and methods

The research protocol is performed in accordance with the relevant guidelines, is in accordance with Declaration of Helsinki, and was approved by the Ethics Committee in Research of Federal University of São Paulo (UNIFESP) under number 1529/2015. This study was conceptualized as primary, experimental, prospective, analytic and comparative design. Informed consent obtained from all patients and/or their legal guardian(s). In this design, 51 pre-molar teeth were obtained from healthy patients through orthodontic extraction procedure. The inclusion criteria for teeth selection were: healthy subjects without chronic use of any kind of drugs, radicular complete formation and signed written consent. Exclusion criteria were sectioned or bleached teeth, dental anomaly or dental whitening^16^.

Premolars were kept at room temperature in Falcon tubes at 10% formaldehyde solution before the use, then washed in de-ionized water, dried at room temperature (24 h). To make easier the access of cleaning substances to pulp chamber, teeth were emerged in a 500 ml of Phosphate Saline Buffer (PBS), with addition of 28 g of EDTA kept at 60C up to translucence (Ph4), then placed in individual recipients for 30 days at room temperature. Second step, were kept 30 days on 9% hydrogen peroxide to clean up the teeth, and final 30 days emerged in enzymatic detergent solution (Riozyme)/distillated water 50% each) to remove the biological residues. They were washed on deionized running water, dried at room temperature for 24 h and packaged in surgical grade paper to be sterilized.

The first tooth was cut is 10 slices (~ 1.5 mm), washed, dried, packed and separated in pairs, these slices were intended to be grinded into powder for analisys at X-Ray Diffractometer (XRD) equipment. The 50 teeth + 10 slices were randomly separated into 5 groups of 10 + 2 slice each, named as groups for decellularization (GD) to avoid direct identification during analysis, and finally submitted to sterilization: DH (group GD1)—170 °C by 120 min; AS (group GD2)—from 60 to 129 °C, and pressure between 1.7 and 1.8 kgf/cm^2^. In two steps, the first 20 min sterilizing, and 30 min drying; EO (group GD3)—packed samples were sterilized through exposition to an ethylene-oxide (30%) + CO_2_ (70%) atmosphere at a relative humidity from 40 to 70%, pressure range 0.4 to 0.5 kgf/cm^2^, exposure time of 4 h with temperature range from 45 to 55 °C. Gamma (group GD5)—packed and sealed samples were exposed to radiation of 25 kGy with multipurpose irradiator of ^60^CO (CTR / IPEN / CNEN-SP), at room temperature and pressure.

The samples were characterized to confirm the preservation of ECM (shape, atomic composition and mechanical resistance) and determine the efficacy of sterilization processes. The teeth were photographed (digital camera cannon model EOS Rebel T100) and radiographed in the longitudinal plane, before and after sterilization processes, using a positioning device to keep uniform distance. On radiographs were used an aluminum scale in order to standardize the different shades of gray observed, where the colorimeter Chroma Meter CR-400—Konica Minolta Camera Co., provided a quantitative measurement to evaluate the color variation after sterilization processes.

To verify the micro-structural interfaces (enamel, dentin, cement and pulp chamber), the samples were carefully sliced, and analyzed at SEM equipment (EVO MA 15 Zeiss equipment), the samples were fixed on stubs and covered with exploded carbon tape, a thin carbon tape established the electrical contact between the sample surface and the sample-holder, allowing the images generation on SEM. The same equipment has an EDS probe included, where was possible to confirm the variation on atomic composition. An additional composition analysis was proceeded using an X-Ray Fluorescence (XRF) equipment, Bruker S2 Ranger, adjusted to analyze a 10 mm diameter and calibrated using pure Cu standard, the equipment has the limitation of analyzing the components through oxide, and Hydrogen is not detectable by the sensors. As the Hydroxyapatite formula is Ca_10_(PO_4_)_6_(OH)_2_ (denoting the crystal unit cell comprises two entities of Ca_5_(PO_4_)_3_(OH)), was decided to confirm the proportionality of Ca (Calcium) and P (Phosphorus) in order to analyze if the sterilization treatments could cause a structural variation caused by temperature, pressure, exposition to ethylene oxide + CO_2_ or gamma irradiation. If existing, this variation could cause an atomic rearrangement crystallographic structure or adsorption of atoms from gas composition of controlled atmosphere (for Example on EO). The samples were ground into powder using pistil and mortar and measured in the XRD equipment D8 diffractometer, with linear position detector type LynxEye from Bruker. The powder was carefully positioned at sample-holder (PPMMA—Poly-methyl-methacrylate) and measured at room temperature under Cu kα radiation, wavelength 1 = 15,406 Å, 2θ scanning from 20° to 120°, step of 0.02° and exposition time of 1.2 s. The data were analyzed through Rietveld Method^40^ at Topas 4.2 software from Bruker AXS, and CIF (Crystallographic Information Files) were obtained from ICSD (Inorganic Crystal Structure Database). Mechanical Resistance—The samples were previously embedded in acrylic resin and polished, and then micro-hardness was measured at Shimadzu HMV 2 T equipment, using 490.3 mN, 0.05 load, and 10 s.

### Sterility efficacy

Microbiological Test—To verify the viability of (anaerobic bacteria was used solution of Thioglycolate (THIO) and for aerobic bacteria, fungi and yeast, was chosen Tryptone Soya Broth (TSB). Negative control sterilization tests were proceeded using two sterilized tubes only with THIO and TSB, The positive tests were performed on 2 inoculated tubes, one only with THIO and the other with TSB solution with no teeth. From the control group GD4 (with no sterilization) one tooth was introduced in the tube with THIO, the second was placed into an inoculated tube with THIO, the third was introduced into a tube with TSB and the fourth into a tube with inoculated TSB. This procedure was done to warranty the cleaning substances residues are not enough to inhibit the microbial growth. The other groups had 2 teeth for each solution (GD1, GD2, GD3 and GD5). All tubes were incubated at 37.5° C for 14 days. The microorganisms were Staphylococcus Aureus (ATCC6538), Bacilus subtilis (ATCC6633), Pseudomonas aeruginosa (ATCC 9027), Clostridium sporogenes (ATCC 11437), Candida albicans (ATCC 10231) and Aspergillus niger (ATCC16404). The concentration was 1 ml for each 15 ml of solution, with 10 to 100 viable microorganisms, in accord to Brazilian Pharmacopeia.

Histology—The dental organs were included in resin block, sliced (2 elements of each group), polished and identified. The samples were colored with Hematoxiline Eosine (HE), Masson Goldner (MG) and Steven Blue (SB), and the pictures were obtained using Inverted Optical Microscopy Axiovert 40C (Carl Zeiss) through Axio Vision 4.1 software.

The data were treated with Kruskal–Wallis (EDS), ANOVA 1 factor (Microhardness) and ANOVA double factor (mass weight losses) tests for statistical validation, in all cases the nullity hypothesis level was set at 0.05 or 5%.

## Results

ECM preservation—The characterization techniques were used to confirm the preservation of ECM in terms of shape, atomic composition, diffractive information about atoms arrangement in the crystalline structure and the micro-hardness of the samples. The mass losses, optical images (photography, radiographies), absence of detected fractures, and minor changes on color suggest the different sterilization methods represents low impact on ECM, supposedly not compromising the scaffolds’ viability.

**Figure 1:**
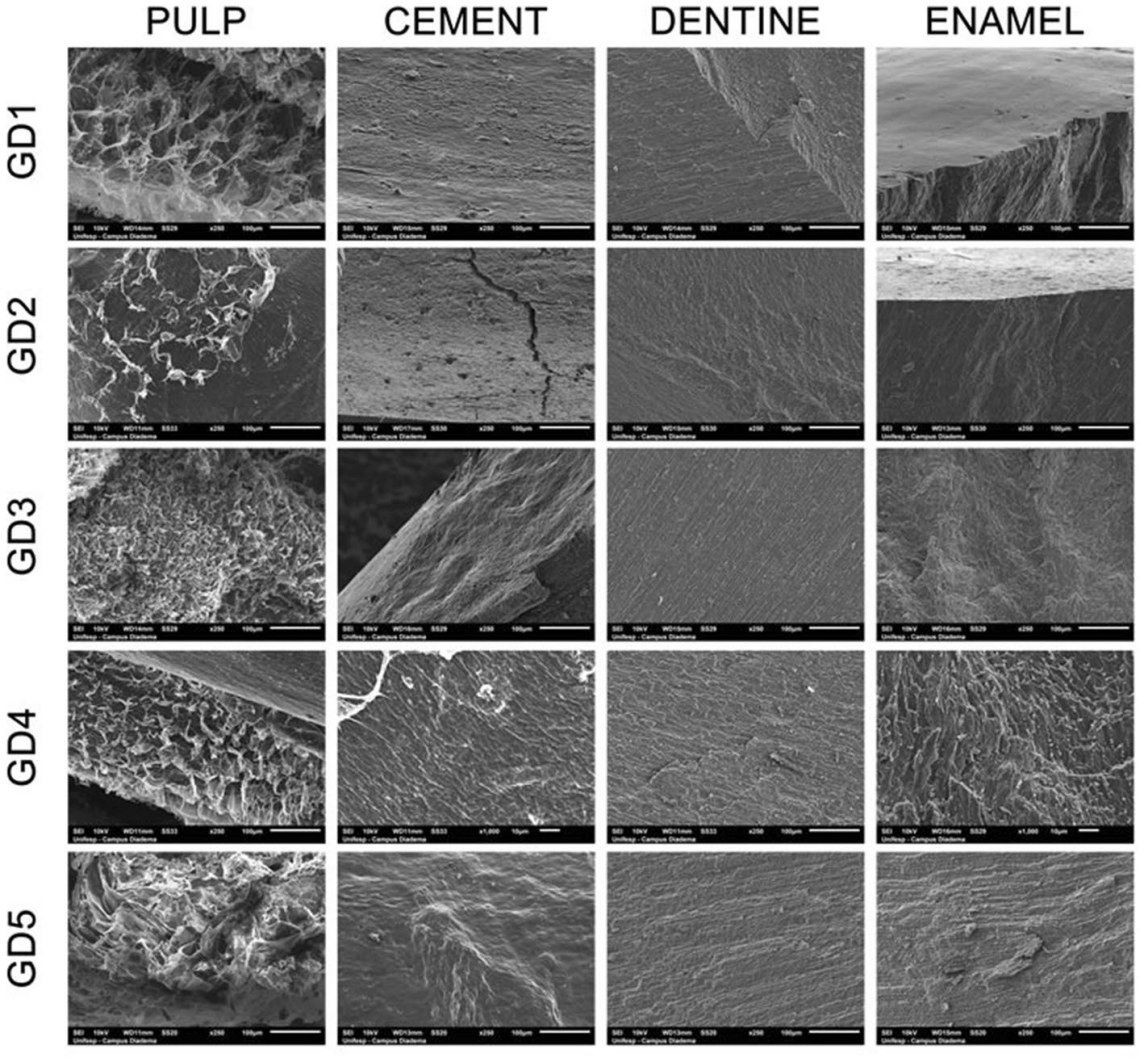
SEM images, obtained at EVO MA ZEISS equipment from UNIFESP, did not evidence visual changes on dentine structure, but presented an apparent smoothing on enamel surface (GD1, GD2, GD3 and GD5), and also some cracks on the cement at GD2 and GD3 (the possibility of being caused by cutting process is not discarded).

As soon the sterilization processes were completed all the teeth were fractioned using sterilized cutting pliers, where was verified presence of pulp in 30% of the control group (GD1) and also on Gamma group (GD5), 20% of DH (GD1) and EO (GD3), and 10% of AS (GD2). Observing SEM’s image, Fig. 1, details of teeth’s structure do not evidence visible variations on hard tissues (cement, dentine and enamel) when comparing the different sterilization processes, however, the same was not observed on soft tissue (pulp). While, sterilization at GD1 and GD5 caused almost no modification, on GD3 and mainly GD2 caused a significant degradation on teeth with remaining pulp.

**Figure 2:**
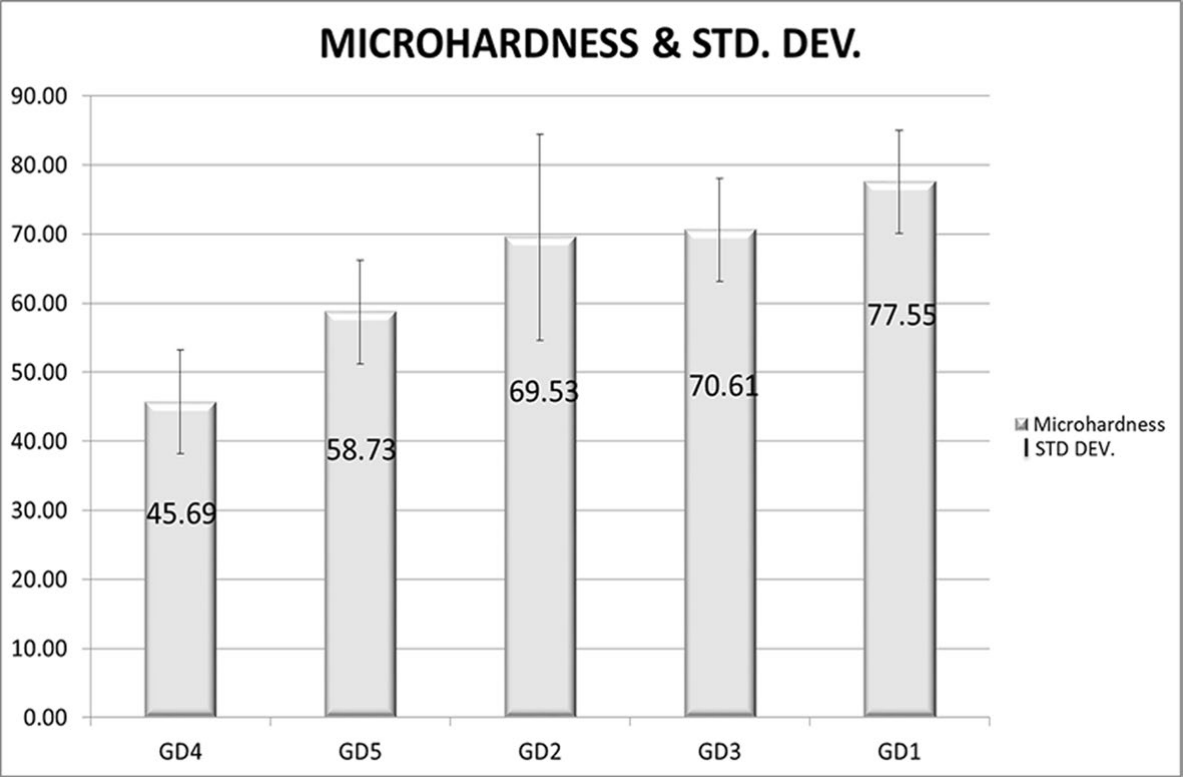
Micro-hardness, measured at SHIMADZU HMV 2T, comparison between control group and sterilization methods, bars indicate the average hardness and the lines represent the standard deviation. Anova statistical analysis (Anova and graphics produced at excel 2013) indicates a significant association between the sterilization method and the micro-hardness, H value is highly significant 52.5432 (*p* = 0.00001).

All sterilization techniques caused significant elevation on hardness, where Gamma Radiation (GD5) is the least impacting, while the most impacting one was dry heat (Fig. 2 presents the comparative measurements). All samples presented small cracking lines, a further study should be interesting to confirm if it would influence the resistance, cells adhesion or even reconstruction of the tissue. Apparently, the temperature has higher influence on hardness, mainly above the water boiling temperature. The control group presented micro-hardness slightly below indicated by Garcia-Garcia^41^ (45.69, varying from 38 to 53HVN), GD5 presented the expected hardness (69.53, varying from 50 to 67HVN), GD2 (69.53 HVN), GD3 (70.61 HVN) and GD1 (77.55 HVN) these results, associated to statistical validation indicates the sterilization method interfere directly on the micro-hardness of teeth, it is important to confirm if this hardness increase can interfere on cell adhesion or not.

**Figure 3:**
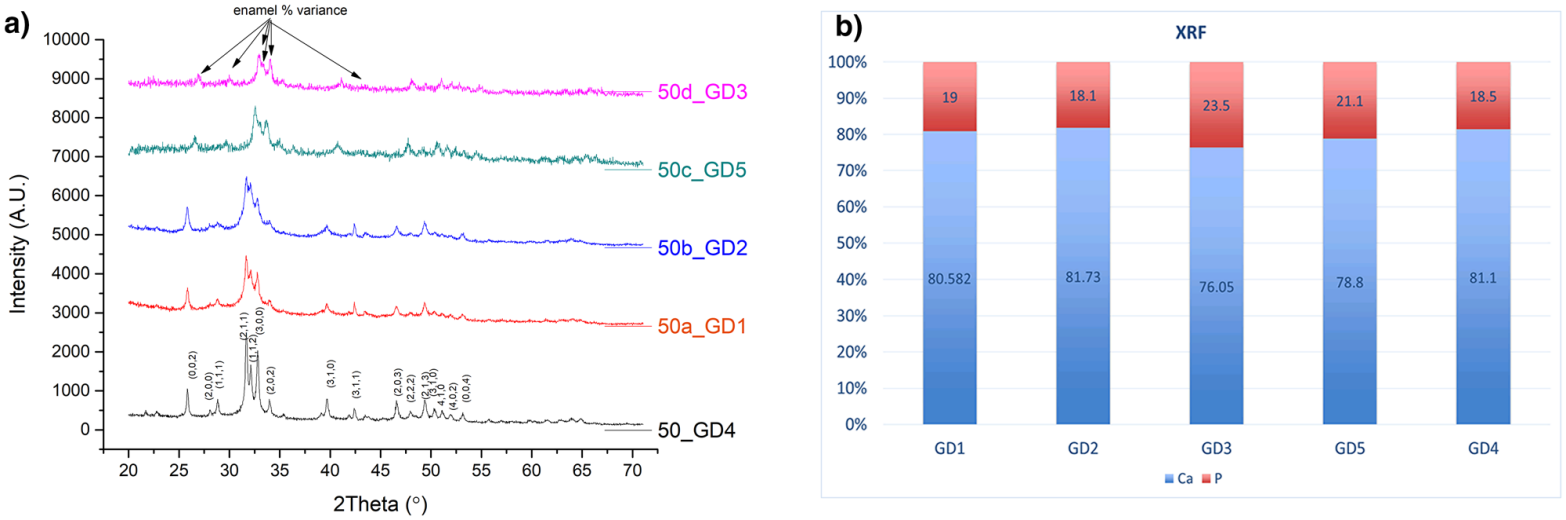
(**a**) Measures at Bruker AXS equipment details the expected angles of the x-ray bean diffraction, CIFs obtained from ICSD—https://icsd.products.fiz-karlsruhe.de/), treated at Topas 4.2 also from Bruker, and plotted using Origin 2015. As it is a complex multi-atomic molecular structure, several angles are represented, but with higher incidence on angles near 26, 32–33, and 40 degrees, the reminiscence of angles positions means no molecular arrangement modification was detected. (**b**) XRF’s data obtained at Bruker’s S2 Ranger equipment the graphic was generated at Excel 2013—the sterilization methods present different effects on the composition balance (Ca/P), indicating GD3 (EO) affects more the Ca portion of Hydroxyapatite and GD2 (DH) interacts more intensively with P portion.

The Rietveld analysis of XRD data suggests the crystalline structures of hydroxyapatite (HA) do not change significantly, although presenting small intensity variation, mainly on angles range of 32–34 degrees; this variation is probably related to different region of sliced teeth analysis (enamel has higher proportion of HA, in sequence are dentine, cement and absent at pulp, the layers were also differently affected by the sterilization process). The reminiscence of peaks on same angles indicates the structural arrangement was not modified, even after the sterilization processes. The refining parameters GOF (Goodness of Fit) 1.40 and weighted profile R-factor (RwP) = 8.51 reinforces the refinement is very close to expected HA’s data. On Fig. 3a) the peaks variation pointed as enamel % variance reflects the technique used, although no change on the 2 theta angles was registered, there was an intensity variation, correlated to higher presence of enamel on some slices. The tooth was cut on crosswise sectioning, where the crown region, with higher presence of enamel, have sharper and more intense peaks than the ones from dentine or cement, in good agreement with reported XRD of enamel and dentine regions^41,42^. The XRF data, Fig. 3b, indicates a small variation on proportion of Calcium (Ca) and Phosphorus (P), being GD3 the more affected, in proportional balance of Ca/P, followed by Gamma Irradiation (GD5), and, apparently, GD1 and GD2 methods did not affect the chemical composition. The hypothesis of structural arrangement modification on HA was not confirmed by XRD analysis, reinforced by the mass losses in every single sterilizing methods (Table 1). Considering the chemical composition of enamel (96% HA and 4% water), dentine (70% HA, 20% organic material and 10% water), and cement (40% HA, 33% proteins and 22% water), the deeper effect of EO (GD3) and Gamma (GD5) were probably because the impact of water, organic material, or slice of tooth analyzed (crown, dentine region or cement).

**Table 1:** Percentage mass losses after sterilization processes.

	Before	After	Δ%
G1	2.0748	1.9882	−4.17
G2	2.1892	2.1278	−2.80
G3	2.0746	2.01239	−3.00
G4	2.0683	2.0683	0.00
G5	2.0126	1.9413	−3.54

The “Table 1” presents the mass variation caused by the sterilization methods, GD4 didn’t varied because was the control group and was not submitted to any sterilization, but also was weighted when the samples were submitted to sterilization and when they returned. Using “Anova double factor without repetition” statistic tool, the different groups presents H = 16.15354 and *p* = 0.009799, meaning a significant association between the masses losses and the method of sterilization.

The EDS information indicates all the sterilizing methods impacted the teeth’s chemical composition (Table 2a–c). The last line of each table is the proportional weight composition of HA, they are very close to GD4 composition on dentine and enamel (justified by the fact dentine has 70% of HA and enamel 90% approximately). The sterilization methods affects more intensively the pulp (Table 2a), probably because this tissue is softer and easier to be modified by pressure or temperature. The significant growth of “others” on GD3, is probably related to adsorption of Ethylene Oxide gas by the pulp; Kruskal–Wallis (KW) statistical analysis: H = 9.217143 and *p* = 0.026539 validate the hypothesis of different sterilization method interfere on the chemical composition. Dentine (Table 2b), has intermediate levels of HA (~ 70%), organic tissue (20%) and water (10%) being less affected than pulp, but here the Oxygen was the most affected compound, although in lower scales the growth of “others” can indicate adsorption of Ethylene Oxide, probably at teeth canaliculated channels; also using KW, H = 16.72686 and *p* = 0.000804 confirms the hypothesis of interference of sterilization method on chemical composition on dentine. Enamel (Table 2c), was the least affected, probably related to the highest level of HA (near 90%), reinforcing the low effect of the sterilization methods on the HA matrix, the same confirmation on Enamel through KW statistic tool, indicates H = 16.71429 and *p* = 0.000809, also confirming the sterilization method interfere on final chemical composition.

**Table 2:** Proportional chemical composition of pulp, dentine and enamel, obtained at EDS equipment, last line of each table indicates expected HA composition. (a) Pulp. (b) Dentine. (c) Enamel.

	Ca	P	O	Others
**(a)**				
GD1	42.30	6.29	50.68	0.73
GD2	48.13	7.38	40.78	3.71
GD3	36.75	8.24	21.26	33.75
GD4	32.71	44.63	19.9	2.762
GD5	42.81	6.36	32.13	18.7
HA	39.89	18.50	41.41	0.20
**(b)**				
GD1	50.59	22.49	26.44	0.48
GD2	59.19	20.6	19.88	0.33
GD3	42.36	20.6	31.69	5.35
GD4	39.17	19.98	38.45	2.394
GD5	46.42	21.43	30.79	1.36
HA	39.89	18.50	41.41	0.20
**(c)**				
GD1	36.6	16.89	39.04	7.47
GD2	46.14	21.09	27.36	5.41
GD3	41.65	19.61	37	1.74
GD4	37.07	18.43	40.03	4.47
GD5	45.69	21.31	31.46	1.54
HA	39.89	18.50	41.41	0.20

**Figure 4:**
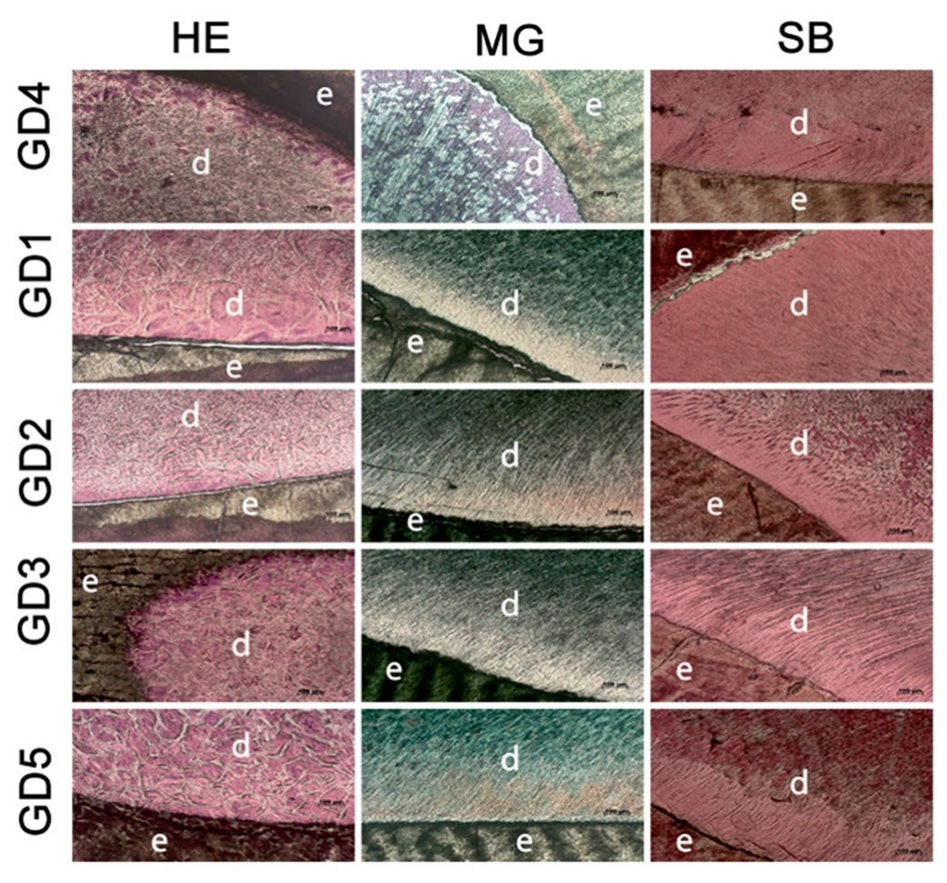
Histology analysis (inverted optical microscopy at AXIOVERT 40C using Axio Vision software)—structural modifications were not identified, although presented a variation on population, in special on GD1, GD2 and GD3 of colorants HE and MG; letter “d” represents dentine, and “e” enamel region. The software used to plot the images was Origin 2015.

Histology images (Fig. 4), using three different standards (Hematoxiline-Eosine (HE), color cell nuclei blue, cytoplasm and collagen fibers pink, the dentine region of GD4 appears darker colored, probably because of miscegenation of nuclei inside the caniculated channels. The sterilized samples presented lighter pink coloration, indicating the sterilization partially eliminated soft tissue, but enamel didn’t present reactive coloration, meaning absence of this kind of cells; Masson Goldner (MG) color cell nuclei black, cytoplasm red and collagen green, the images indicate higher concentration of collagen on dentine region, in special on dentine of GD4 and GD5 groups, enamel didn’t present reactive coloration; and Stevenel’s Blue (SB) color in blue collagen and fibers, and red-pink the calcified tissues; The coloration at dentine and enamel evidenced calcified structure, not being possible to affirm nuclei or soft tissue are present, dark dots and lines are apparently the canaliculated channels. The images with HE and MG colorants suggests GD1, GD2 and GD3 were more efficient on removing soft tissue from the teeth.

Although the sterility tests are very well known techniques, with large application to several materials, their usage on dental scaffolds is not frequently reported, to reduce the possibility of rejection of these scaffolds the sterilization methods are the central core of this research. Here, the negative control (NC) test, with THIO and TSB solutions, confirmed there were no contamination on solutions or tubes. Two empty tubes were inoculated, and presented microorganisms proliferation, showing the effectiveness of THIO and TSB solutions. In sequence were run the positive control (PC) tests, preparing 20 falcon tubes, 4 for each sterilized group and control group (GD1, GD2, GD3, GD4 and GD5). The absence of microorganisms, after incubation process, evidenced the efficacy of each sterilization method. Indeed, the control group (GD4) and the two empty tubes also were inoculated (PC and NC), presenting proliferation of microorganisms, validating the hypothesis the samples and solutions were not contaminated, the active actuation of THIO and TSB solutions and effectiveness of the sterility tests. The results are reported on Table 3.

**Table 3:** Sterility test—the signals “ + ” indicates the presence of microorganisms, and “−“ absence, PC and NC are abbreviations of “Positive Control” and “Negative Control”.

	TIO	TSB
Negative CRTL (NC)	−	−
Positive CTRL (PC)	+	+
Inoculated PC	+	+
Inoculated NC	+	+
Control group (GD4)	+	+
Dry heat (GD1)	−	−
Autoclave (GD2)	−	−
Ethylene oxide (GD3)	−	−
Gamma radiation (GD5)	−	−

## Discussion

The use of sterilization procedures on medical or dental devices is a common practice, even on artificial implants as titanium teeth implants, the study of Manea^43^ provided evidences about the efficacy of moist heat (autoclave) and dry heat sterilization procedures, being compatible to results obtained in this research. These two techniques are standardized on ISO17665-1:2006 and ISO 17665–2: for moist heat and ISO 20857:2010 for dry heat, even Ethylene Oxide (ISO 111135:2014) or irradiation (ISO 11137–2:2013) are standardized, but up to now, was not found reports to their application on dental teeth intended to be used as scaffolds.

Femoral heads, processed bones, tendon or skin are good examples of allograft tissues used on transplantation or functional recovery surgeries, Singh^44^ reports the importance of sterilization when proceeding these tissues, some methods cited include moist heat (autoclaving), ethylene oxide and gamma irradiation. The study does not recommend ethylene oxide because of a toxic gas production (ethylene chlorohydrin) what could cause synovial inflammation, and focuses on gamma irradiation. Fölsch^45^ studied the thermo-disinfection on cancellous bones reporting the tensile strength was preserved, agreeing to supposed preservation of structural resistance on ECM.

In this study, after partial decellularization process, almost 30% of samples still presented biological residues at the pulp chamber, repeating the results previously obtained on the partial demineralization study^31^. When submitted to sterilization, the results suggest the influence of temperature and pressure variation on reduction of remaining pulp. Except by GD5 process, all the others presented a significant reduction; GD5 was run at room temperature (~ 20 °C) and pressure (~ 1kgf/cm2) still presented 30% of remaining pulp; GD1 at room pressure and 170 °C, reduced from 30 to 20%; GD3, at pressure between 0.4 and 0.5 kgf/cm2 and temperature 45–50 °C, also reduced from 30 to 20%; GD2, with pressure between 1.7 and 1.8 kgf/cm^2^ and temperature from 60 to 129 °C, reduced from 30 to 10%, indicating the combination of higher pressure and temperature have stronger impact on the reduction of remaining pulp.

As the aim of this research was to evaluate the four sterilization methods, could be said the four methods were well succeeded, as all of them are suitable to produce sterilized scaffold. None of methods impacted on structural molecular arrangement (XRD), although, caused impact on chemical composition (XRF and EDS), and also caused higher micro-hardness. Considering the cost–benefit criteria associated to reduction of remaining pulp, the results suggest the Autoclave Sterilization (GD2) has some advantages in terms of competitive appeal. However, a deeper study to confirm the compatibility with stem cells should be proceeded previously to discard dry heat (GD1), ethylene oxide (GD3) or gamma radiation (GD5).

## Conclusion

The results evidenced the decellularization alone is not enough to eliminate microorganisms from dental scaffolds, and the four sterilization methods were fully effective with preservation of ECM molecular arrangements, but with some variation on chemical composition (mainly proportion of Ca/P). Gamma irradiation (GD5) and ethylene oxide (GD3) presents excellent results, but their viability are compromised by the costs and mainly the technology’s accessibility (requires very expensive equipment and/or consumables), exception to gamma irradiation (GD5), all the others also reduced the remaining pulp. Autoclave (GD2) presented easy equipment accessibility (with lower cost consumables), higher reduction of remaining pulp and complete sterilization, reason why was considered the most promising technique.
